# Supplementary material for: The diagnostic value of serum insulin-like growth factor binding protein 7 in gastric cancer
Source: PeerJ. 2023 Jun 5;11:e15419. doi: 10.7717/peerj.15419 (PMC10249617; doi:10.7717/peerj.15419)

Supplementary Figure S1. The expression heatmap of IGFBP-rPs in TCGA. Yellow meant the high expression while blue meant the low expression.

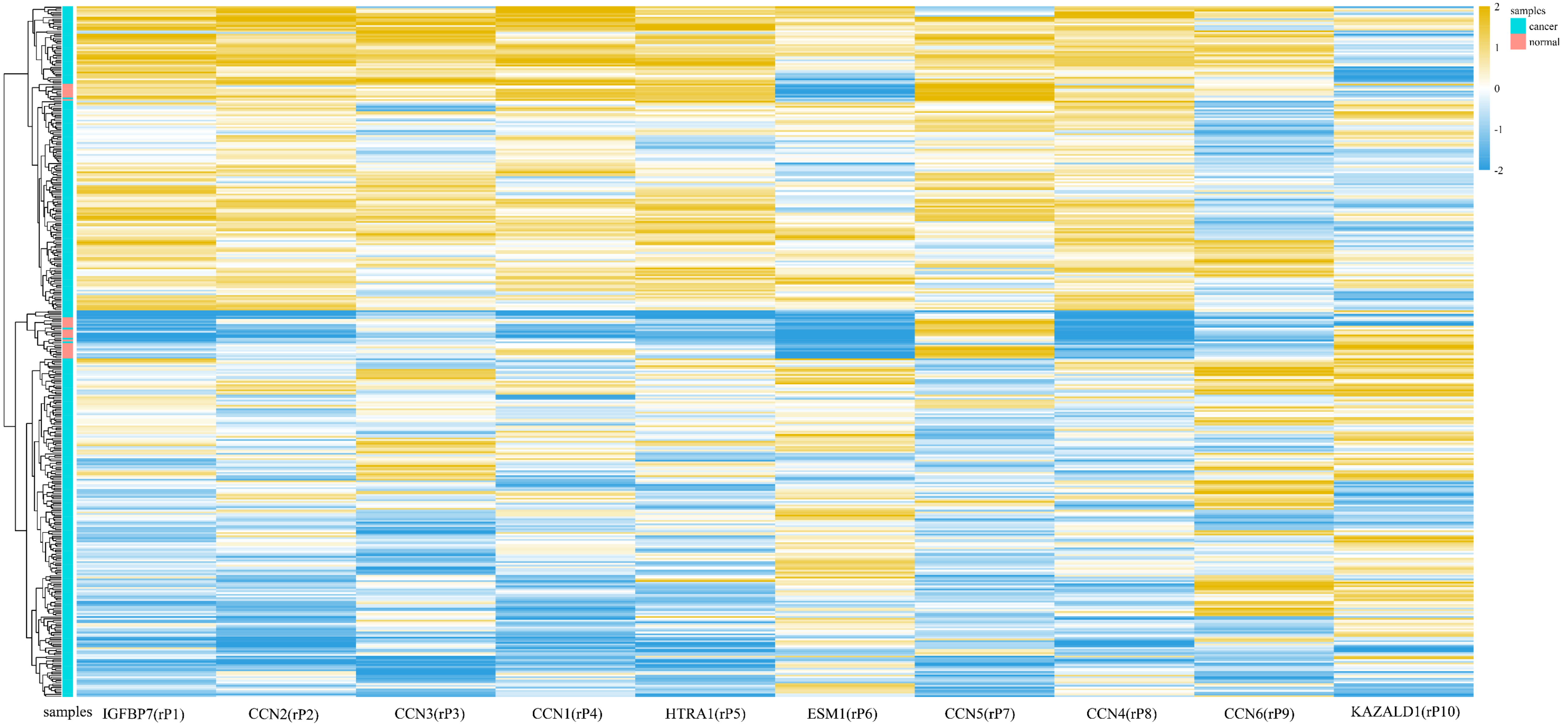

Supplementary Figure S2. The expression level of IGFBP-rPs in gastric cancer tissues and adjacent normal tissues.

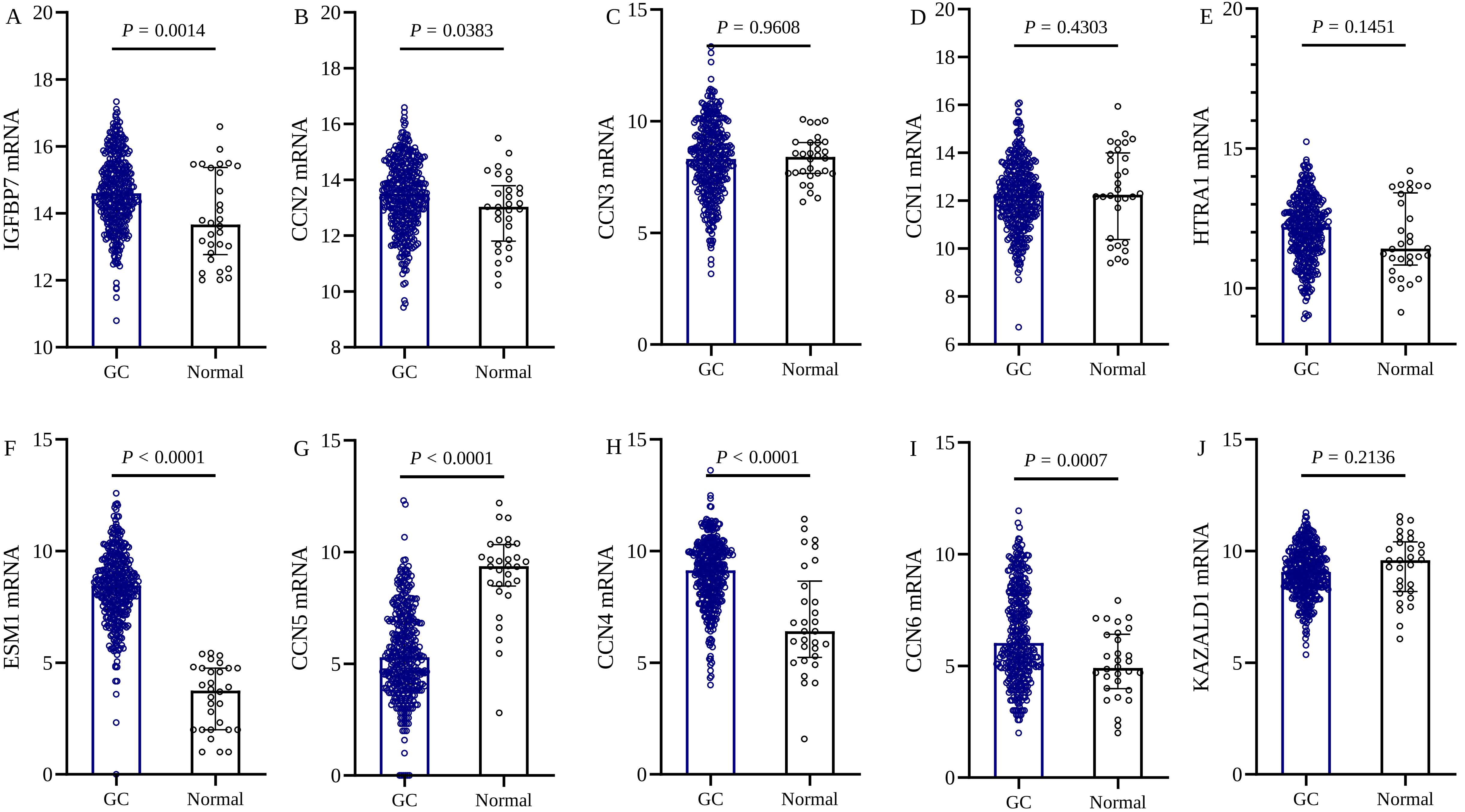

Supplementary Figure S3. Kaplan–Meier survival analysis to assess the association between IGFBP-rPs and overall survival of gastric cancer patients in TCGA.

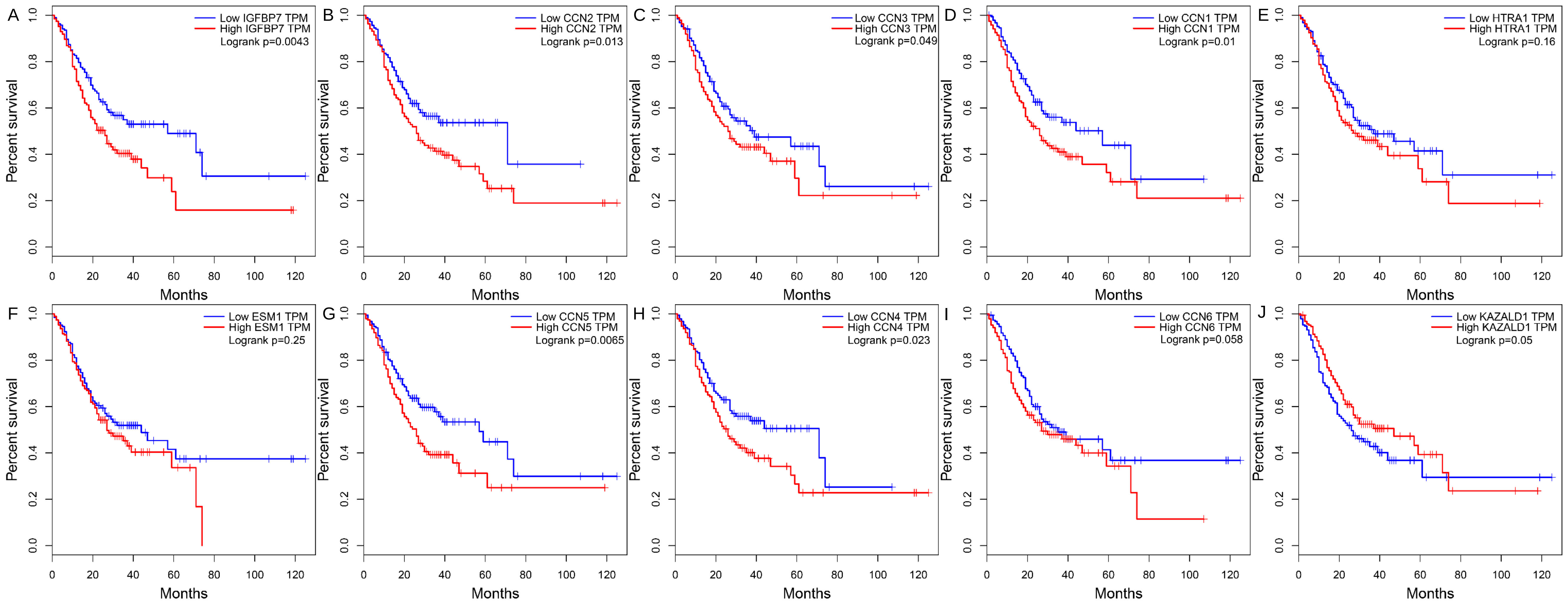

Supplementary Figure S4. Kaplan–Meier survival analysis to assess the association between IGFBP-rPs and disease-free survival of gastric cancer patients in TCGA.

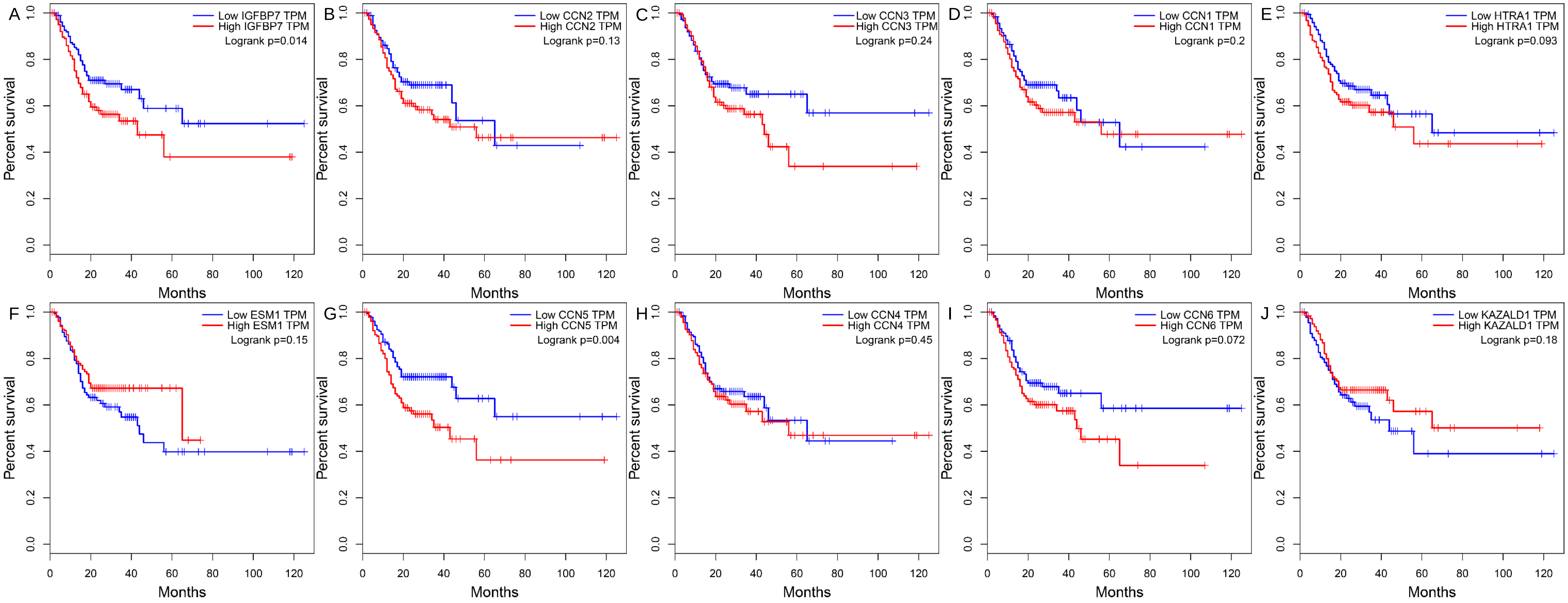

Supplementary Figure S5. Correlation between serum IGFBP7 level and clinical characteristic in training (A) and independent validation (B) cohorts.

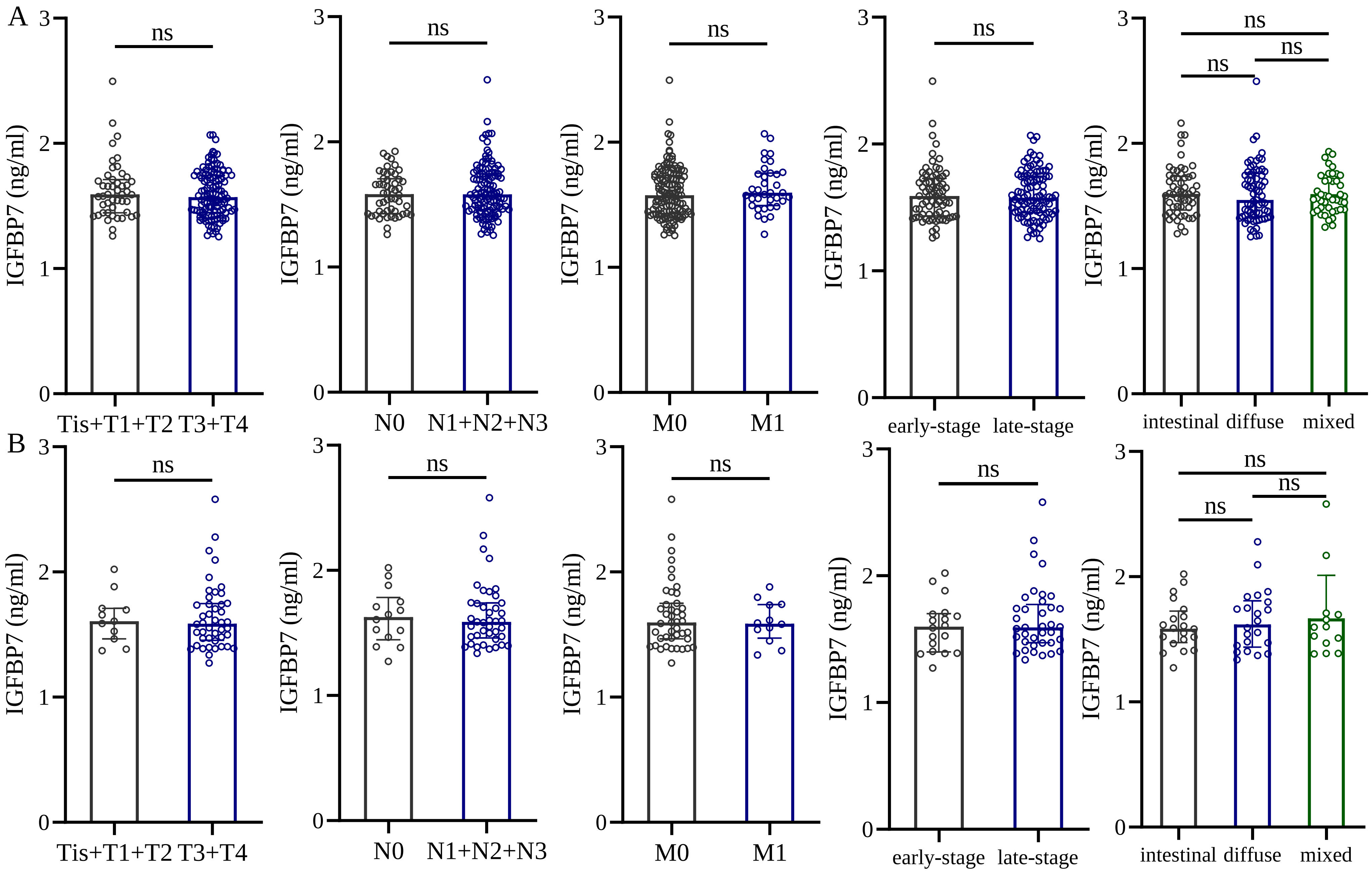

Supplementary Figure S6. The comparison of serum IGFBP7 level in male and female separately.

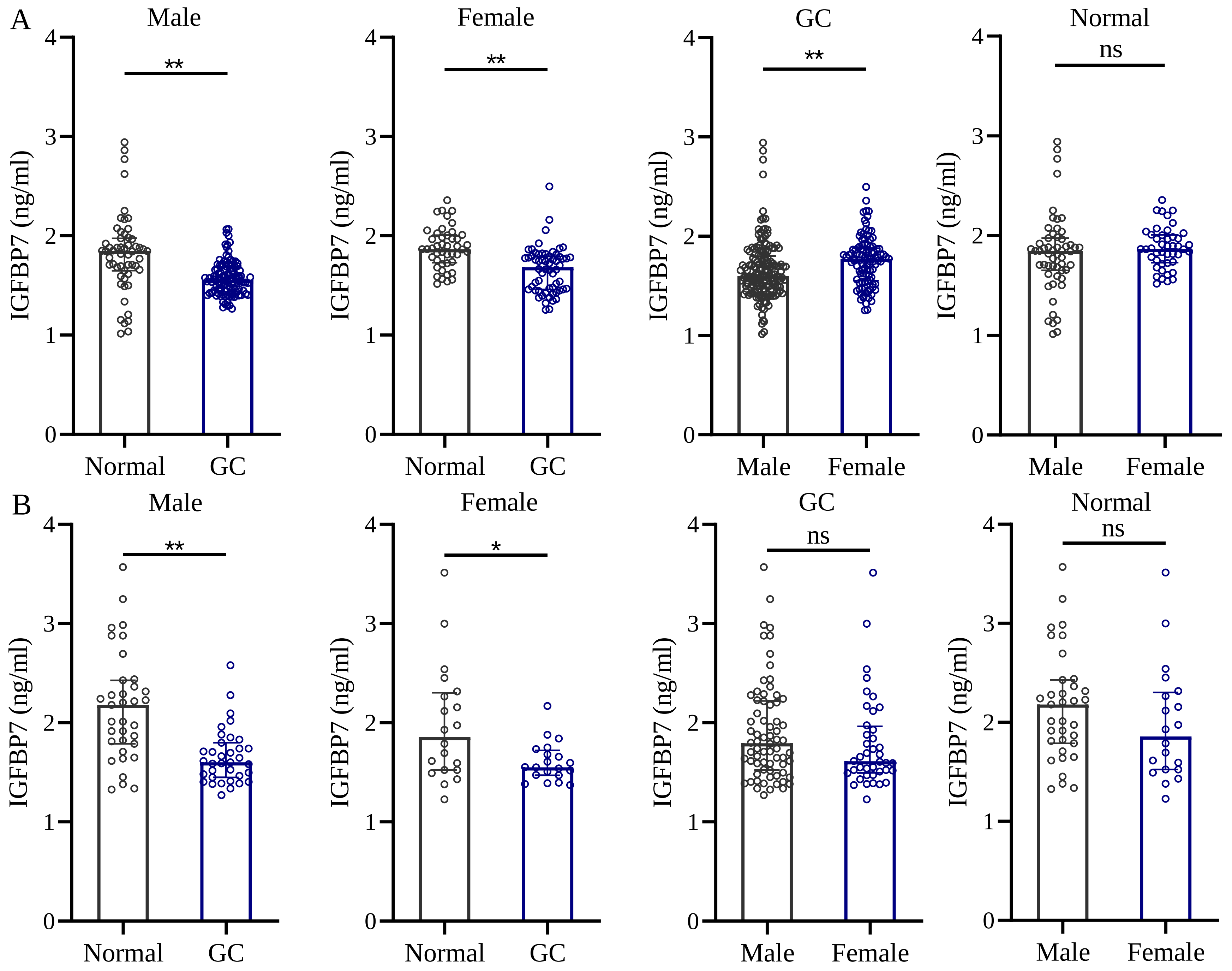

Supplementary Figure S7. The expression of IGFBP7 mRNA level in pan-cancers using TCGA and GTEx database.

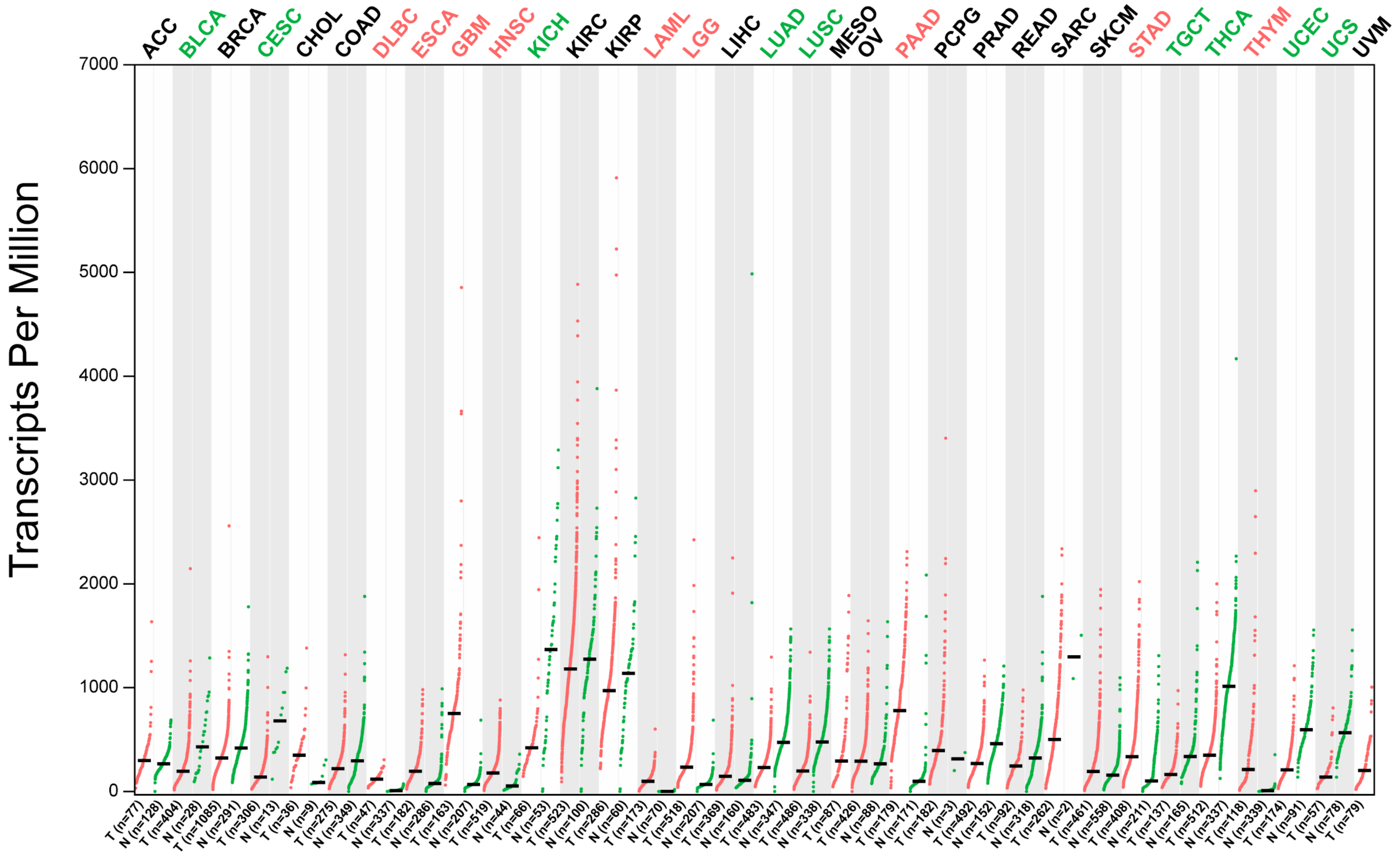

Supplement: Supplemental Information 2 [file peerj-11-15419-s002.pdf]
